# Closed Traumatic A2 Through A4 Pulley Rupture and Flexor Digitorum Superficialis Avulsion Treated With Reconstruction

**DOI:** 10.31486/toj.19.0109

**Published:** 2021

**Authors:** Bhumit Desai, Gonzalo Sumarriva, Ross Dunbar

**Affiliations:** ^1^ Department of Orthopedic Surgery, Ochsner Clinic Foundation, New Orleans, LA; ^2^ The University of Queensland Faculty of Medicine, Ochsner Clinical School, New Orleans, LA

**Keywords:** *Hand injuries*, *reconstructive surgical procedures*, *tendon injuries*, *trigger finger disorder*

## Abstract

**Background:** Multiple closed spontaneous pulley ruptures are rare injuries and require surgical reconstruction to prevent functional deficits. Pulley rupture combined with avulsion of the flexor digitorum superficialis (FDS) tendon is an even more uncommon occurrence.

**Case Report:** We describe a closed traumatic annular 2 (A2) through annular 4 (A4) pulley rupture with avulsion of the FDS tendon. This uniquely associated pathology was treated with a complex surgical reconstruction that corrected flexion contracture and tendon bowstringing in the left long finger. The desired outcome was achieved through A2 and A4 pulley reconstruction using an autologous palmaris longus tendon graft with FDS tendon excision and proximal interphalangeal joint capsulotomy.

**Conclusion:** Multiple pulley rupture is not commonly combined with FDS avulsion, and treatment of this injury requires careful surgical planning based on pulley biomechanics to maximize postoperative function.

## INTRODUCTION

Closed flexor tendon pulley ruptures are especially frequent among rock climbers but can also happen through daily activity.^1-4^ Most of these injuries occur as isolated single-pulley ruptures of either the annular (A) or cruciate (C) pulleys.^5^ Multiple combined pulley ruptures are rare and require surgical reconstruction to prevent functional deficits.^3,6^

The finger flexor pulley system of the second to fifth fingers is composed of 5 annular (A1-A5) and 3 cruciate (C1-C3) pulleys. The A2 pulley is located on the proximal half of the proximal phalanx, the A3 pulley arises from the volar plate of the proximal interphalangeal (PIP) joint, and the A4 pulley is located at the midportion of the middle phalanx. The pulleys function to keep the flexor tendons close to the bone, allowing a translational force to be directed by the flexor muscle-tendon unit and resulting in rotational movement of the phalanges.^7^

Compounding the rarity of such pulley injuries are associated avulsions of the flexor digitorum superficialis (FDS) tendon.

To our knowledge, this report presents only the third case describing the combined injury of a closed traumatic FDS avulsion with flexor tendon pulley ruptures.^8,9^

## CASE REPORT

A 58-year-old right-hand-dominant male who worked as a diesel mechanic presented to the hand clinic with 1 month of pain and decreased range of motion in his left long finger after the finger was hyperextended when he lifted a heavy brick stepping-stone. His examination was notable for flexion contracture of the left long finger at the PIP joint. X-rays were unremarkable for osseous or soft tissue abnormality. He was treated conservatively with a dynamic splint and physical therapy.

**Figure 1.**
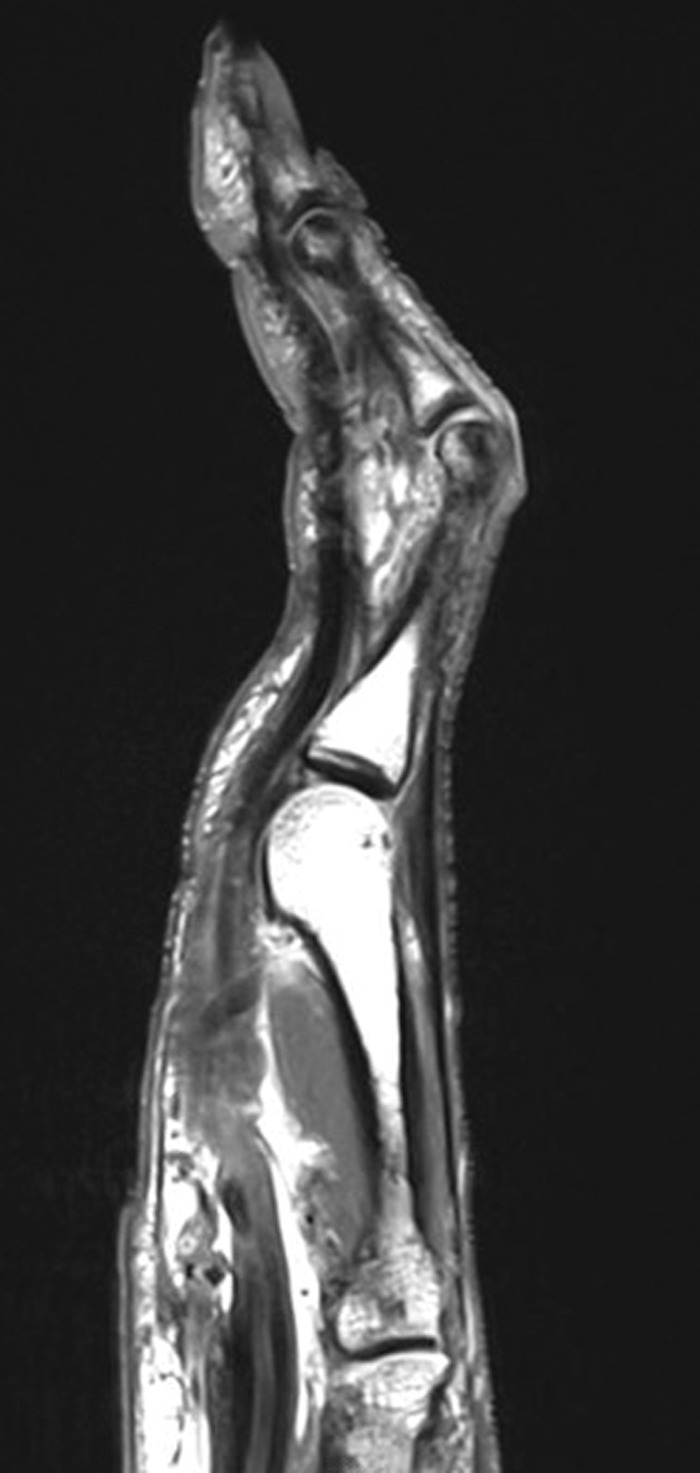
**Sagittal proton density magnetic resonance imaging demonstrates complete ruptures of the annular 2 (A2) pulley and flexor digitorum superficialis tendon of the long finger. Bowstringing of the flexor digitorum profundus tendon is suggestive of concomitant annular 3 (A3) pulley rupture.**

Six months later, the patient sought a second opinion after gradual worsening of his symptoms. Examination revealed progressive worsening of the flexion contracture with bowstringing of the left long finger on resisted PIP joint flexion. The left long finger had a flexion contracture of 55 degrees, with PIP joint range of motion limited to 60 to 100 degrees of flexion. The examination was notable for no independent function of the FDS tendon. Repeat x-rays remained unremarkable. Ultrasound showed the proximal stump of the FDS tendon at the level of the metacarpal head, and magnetic resonance imaging (Figure 1) demonstrated complete ruptures of the A2 pulley and FDS tendon, with bowstringing of the flexor digitorum profundus (FDP) tendon, suggestive of an A3 pulley rupture. After failing another round of conservative management, the patient elected to undergo reconstruction. The risks, benefits, and alternatives to surgery were discussed with the patient, and informed consent was obtained. The time from injury to surgical repair was 9 months.

The left long finger was incised using an extensive Bruner incision from the mid-palm to the fingertip. Traction on the FDP tendon confirmed bowstringing over the PIP joint, and the surrounding scar tissue was excised. The retracted FDS tendon was identified in the midpalm and was surrounded by significant scarring. The decision was made to harvest the palmaris longus tendon. The FDS tendon was resected after its identity was confirmed through the palmaris harvest incision. Deeper dissection revealed scar tissue around the FDP tendon in the middle and proximal phalanges that required resection. A left long finger PIP joint capsulotomy with partial excision of the volar plate, checkrein ligaments, and collateral ligaments was undertaken to correct the flexion contracture and treat the patient's inability to passively extend the left long finger.

**Figure 2.**
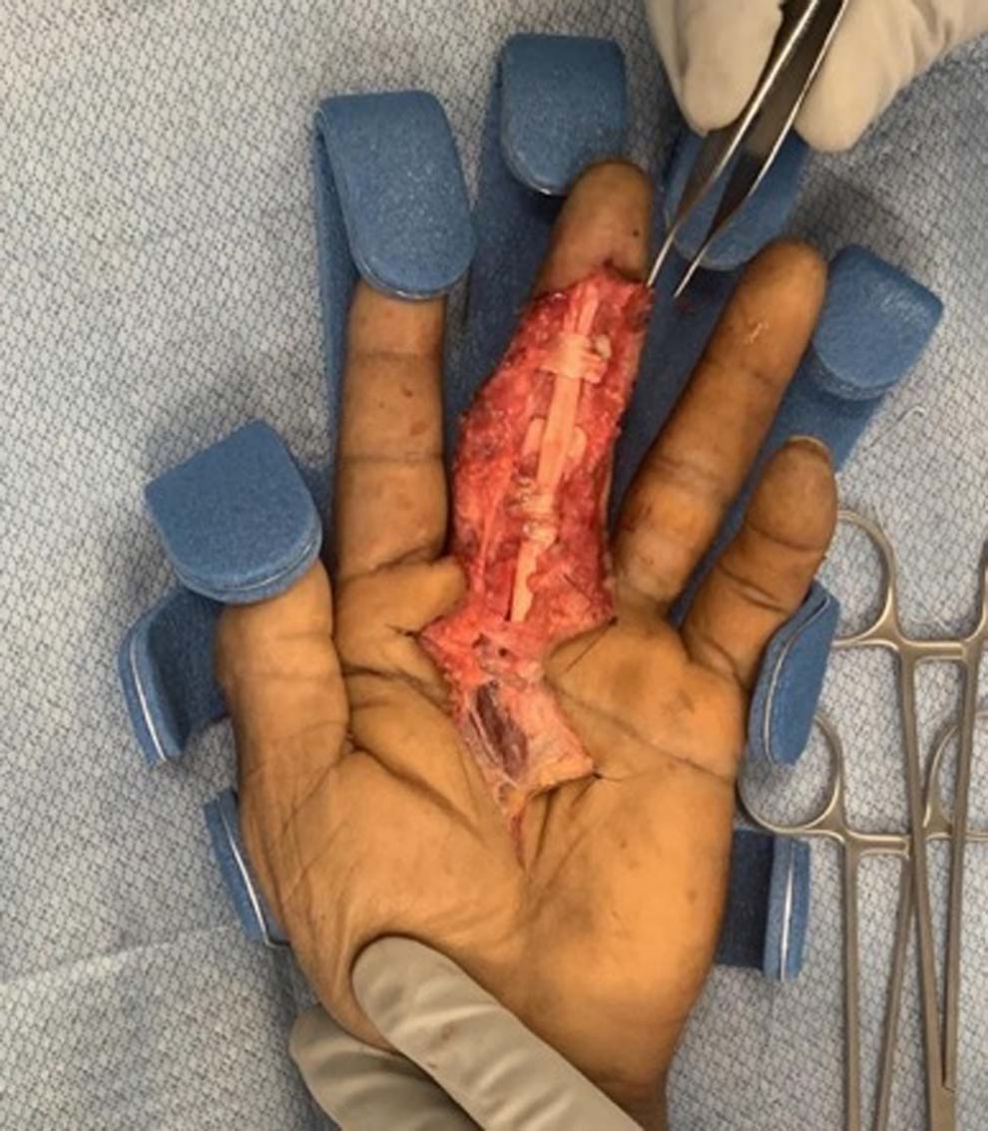
**Intraoperative photograph demonstrates the annular 2 (A2) pulley reconstruction with autologous palmaris longus tendon and the annular 4 (A4) pulley reconstruction with autologous flexor digitorum superficialis tendon.**

Pulley reconstruction started with the ruptured A2 pulley. The harvested palmaris longus tendon was looped 3 times around the proximal phalanx in the region of the original pulley and then tensioned appropriately with the distal end sutured to itself. The bowstringing improved, with smooth gliding of the FDP tendon confirmed at the A2 pulley site. Because of some residual bowstringing in the region of the deficient A4 pulley, the surgeon decided to reconstruct the A4 pulley. A viable portion of the resected FDS tendon was split down the middle and passed twice around the middle phalanx. After securing the distal end to itself, smooth gliding of the FDP with correction of bowstringing was confirmed (Figure 2). Full passive extension was achieved. After surgery, the patient was positioned in a short-arm volar splint to the left upper extremity with the fingertips in full extension to minimize stress on the newly reconstructed pulleys.

Six weeks postoperatively, the patient's flexion contracture of the left long finger PIP joint had fully resolved. Range of motion of the left long finger metacarpophalangeal joint was nearly full. Passive range of motion of the PIP and distal interphalangeal (DIP) joints was 55 degrees and 40 degrees, respectively (Table 1). Active range of motion of the PIP and DIP joints was limited to approximately 5 degrees each.

**Table 1.** Range of Motion (ROM) Progression From Presentation to 3-Month Follow-Up

Joint/ROM	Preoperatively	6 Weeks Postoperatively	3 Months Postoperatively
Proximal interphalangeal joint			
Joint flexion contracture	Present	Resolved	Resolved
Passive ROM, degrees	60-100	0-55	0-50
Active ROM, degrees	0	0-5	0-20
Distal interphalangeal joint			
Passive ROM, degrees	0	0-40	25-50
Active ROM, degrees	0	0-5	25-40

Three months postoperatively, the patient had developed significant stiffness in extension of the left long finger. Because the pain had completely resolved and partial functionality had returned, the patient reported satisfaction with the procedure and elected to forego further treatment. He was advised to follow up as needed for deterioration in function or to discuss potential interventions such as tenolysis or PIP joint arthrodesis.

## DISCUSSION

The standard treatment for single pulley ruptures is conservative management; however, management principles change drastically when multiple pulley injuries are involved.^3,6^ The role of the flexor pulley system is to maintain close contact between the flexor tendons and the phalanges, thus applying a short moment arm across the PIP joint.^10^ Subsequent tendon bowstringing results in a loss of tendon excursion, loss of active flexion, and the potential for PIP joint flexion contracture.^11,12^ Biomechanical studies have shown that the A3 pulley alone is of little importance in preventing tendon bowstringing at the PIP joint.^13^

A review of pulley reconstruction techniques yields several approaches, differing by the number of fastening loops and/or autologous graft source. The Weilby technique has been shown to produce excellent functional outcomes,^14^ while the Widstrom loop-and-a-half technique is the strongest repair.^12^ Bunnell^15^ was effective with the single-loop technique, while Okutsu et al^16^ and Mehta and Phillips^17^ opted for the triple-loop technique. Doyle and Blythe^18^ argued for the palmaris longus tendon as the ideal autologous graft, Lister^19^ described repairs using the flexor retinaculum, while Gabl et al^6^ and Moutet^20^ preferred the extensor retinaculum. Karev et al^21^ described an altogether unique belt-loop approach involving 2 transverse incisions in the volar plate.

To our knowledge, this case is only the third report of combined pulley rupture and FDS avulsion (Table 2). The first case was reported in 2001 by Vandeputte and Dubert, who described a 48-year-old male who ruptured the A2 through A4 pulleys with an avulsed FDS tendon of the right ring finger while forcefully making a fist.^8^ Three months after the initial injury, the patient underwent surgical reconstruction that involved an autologous palmaris longus tendon graft. The patient experienced significant improvements in range of motion after the repair, with a full return to activities by 5 months.^8^ The second case was reported in 2014 by Johnsen et al.^9^ They described a 56-year-old male who presented with pain and swelling of the right long finger resulting from release of a bowling ball. Ultrasound confirmed A2 and A3 pulley ruptures with avulsion of the FDS tendon. Seventeen days after the injury, the patient underwent surgical reconstruction of the A2 and A3 pulleys according to the Weilby technique using an autologous palmaris longus tendon. He had increased range of motion by 2 months postoperatively and achieved a full return to activity at 15 months.

**Table 2.** Cases Describing Combined Pulley Rupture and Flexor Digitorum Superficialis (FDS) Avulsion

Variable	Vandeputte and Dubert, 2001^8^	Johnsen et al, 2014^9^	Current Case
Age, years/sex	48/M	56/M	58/M
Occupation	Office worker	NR	Diesel mechanic
Digit involved	Right ring	Right long	Left long
Hand dominance	Right	NR	Right
Inciting event	Contact against closed fist	Releasing bowling ball	Lifting brick
Pulleys involved	A2, A3, A4	A2, A3	A2, A3, A4
FDS retraction	Distal palmar crease	Proximally	Midpalm
PIP flexion contracture, degrees	50	Not present	55
Preoperative PIP ROM, degrees	50-80	0-70	60-100
Time to surgery	3 months	17 days	9 months
FDS resected?	Yes	Yes	Yes
Pulleys reconstructed	A2 (PL), A4 (PL)	A2 (PL Weilby), A3 (PL)	A2 (PL triple loop), A4 (split FDP double loop)
Postoperative PIP active ROM, degrees	20-85	0-90	0-5
PIP active ROM at longest follow-up, degrees	10-90 (5 months)	10-100 (15 months)	0-20 (3 months)

A2, A3, A4, annular 2, annular 3, annular 4 pulleys; FDP, flexor digitorum profundus; M, male; NR, not reported; PIP, proximal interphalangeal joint; PL, palmaris longus tendon graft; ROM, range of motion.

In our case, the surgeon opted for the triple-loop technique to prevent further bowstringing of the FDP tendon. The reconstruction was tested and shown to have smooth gliding of the FDP with correction of the flexion contracture. The A3 pulley was not reconstructed because of its anatomic location over the joint. A reconstructed A3 pulley would have a minimal biomechanical role in preventing further bowstringing of the FDP tendon. In cases of concomitant FDS and pulley loss, repair of the FDS avulsion has been deemed to be unnecessary because it would likely impede gliding of the FDP through zone II.^9^ Zone II refers to the area between the FDS insertion and the distal palmar crease, as described in the classification system of flexion tendon injuries by Verdan in 1960.^22^

Patients with preexisting PIP joint flexion contractures are associated with less favorable functional outcomes compared to patients who undergo reconstruction before developing contractures.^8^ Contractures in pulley injuries arise from an increased lever arm at the PIP joint. Additionally, scar tissue impedes contracture resolution after pulley repair. As seen in our patient, contracture release achieved through PIP joint capsulotomy and release of the checkrein ligaments and the collateral ligaments led to full passive range of motion at the PIP joint. Such releases should be managed with postoperative dynamic extension splinting to reduce the risk of contracture recurrence.

A rapid extension force acutely applied to a flexed finger has been described as the mechanism of injury in pulley ruptures by Bowers et al.^11^ Marco et al conducted cadaver experiments in which the flexor tendons were loaded until pulley and tendon rupture occurred, at which point they hypothesized that a transfer of force from the bowstringing profundus tendon to the superficialis led to avulsion of the FDS tendon.^23^ The clinical progression of our patient from mechanism of injury to symptoms at time of presentation is consistent with similarly described cases. Compared to previously reported cases of combined pulley rupture and FDS avulsion, our patient had a worse outcome in postoperative range of motion. Timing of the reconstruction was an important factor in the patient's outcome, with 9 months from injury being less than ideal. Ultimately, the patient traded a stiff finger in flexion for a stiff finger in extension. The patient was satisfied with this new position as he stated he is more functional in daily life, such as being able to place his hands in his pockets.

## CONCLUSION

FDS tendon avulsion combined with multiple pulley rupture is a rare combination of pathology that requires a systematic approach to diagnosis, with treatment focused on maximizing patient function. Knowledge of flexor pulley system anatomy, the mechanism of injury, and the clinical presentation based on severity of injury can help guide management principles and expectations after reconstruction.

## Notes

This article meets the Accreditation Council for Graduate Medical Education and the American Board of Medical Specialties Maintenance of Certification competencies for Patient Care and Medical Knowledge.
